# Down Regulation of ackA-pta Pathway in *Escherichia coli* BL21 (DE3): A Step Toward Optimized Recombinant Protein Expression System

**DOI:** 10.5812/jjm.8990

**Published:** 2014-02-01

**Authors:** Nahid Bakhtiari, Manouchehr Mirshahi, Valiollah Babaeipour, Nader Maghsoudi, Abbas Tahzibi

**Affiliations:** 1 Biochemistry Department, Faculty of Biological Sciences, Tarbiat Modares University, Tehran, IR Iran; 2 Department of Bioscience and Biotechnology, Malek Ashtar University of Technology, Tehran, IR Iran; 3 Neuroscience Research Center, Shahid Beheshti University of Medical Sciences, Tehran, IR Iran; 4 Food and Drug Organization, Ministry of Health of Iran, Tehran, IR Iran

**Keywords:** *Escherichia coli*, Acetate, Antisense RNA, Down-Regulation

## Abstract

**Background:**

One of the most important problems in production of recombinant protein is to attain over-expression of the target gene and high cell density. In such conditions, the secondary metabolites of bacteria become toxic for the medium and cause cells to die. One of these aforementioned metabolites is acetate, which enormously accumulated in the medium, so that both cell and protein yields are affected.

**Objectives:**

To overcome this problem, several strategies applied. In this research we used antisense RNA strategy, where the transcription of phosphotransacetylase (PTA) and acetate kinase (ACK), two acetate pathway key enzymes, could be controlled, which led to reduced acetate production.

**Materials and Methods:**

In order to achieve this, recombinant plasmid harboring antisense sequences targeting both of *pta* and *ackA* was assembled, after transfecting to the cells, its effects on the cell growth and acetate accumulation in the minimal media was assessed and compared with the control, the plasmid without antisense cassette, in presence and absence of IPTG in *Escherichia coli* BL21 (DE3).

**Results:**

It was observed that the mentioned strategy partially affect the growth and amount of excreted acetate in comparison with the control. In addition it was found that high down-regulation of the acetate production pathway reduces the growth rate of *E. coli* BL21 (DE3).

**Conclusions:**

The study principally proved the importance of this strategy in acetate excretion control.

## 1. Background

*Escherichia coli* is employed for production of great amounts of small size proteins and have a quite uncomplicated structure in industry. To have high cell density and protein yield, fed-batch cultivation in a fermanter is generally applied (1). However, acetic acid excretion is a problem in fed-batch fermentations and decreases the recombinant protein yields (2-5). Acetogenesis occurs because the cell needs to regenerates the NAD+, which is used by glycolysis and to recycle the coenzyme A required for converting of pyruvate to acetyl-CoA (6).

Different approaches have been suggested to investigating the decrease of acetic acid accumulation. Most of these strategies is rooted in one of the subsequent classes: prevention of dissolved oxygen deficiency by increasing the agitation speed or improving the pure oxygen; limiting the concentration of nutrient of cell by regulating the medium feed rate using complicated control algorithms for fed batch process; elimination of toxic waste, particularly acetic acid from used medium by *in situ* machines, such as a perfusion system. However these approaches can be resulted to a better performance, their achievement is extremely hard because the working plan to attain best outcomes requires very accurate control in addition to expensive costs (7). In addition, “knockout” mutations inhibit acetic acid production ignore that the acetate pathway have a significant physiological function on *E. coli* (8).

Recently, antisense RNA technology has provided a novel genetic manipulation method to decrease the unfavorable effects of acetic acid production (8). Antisense RNA is an ssRNA that has a complementary sequence of a mRNA transcribed in a cell, and may be entered into a cell to restrain translation of a target mRNA by hybridization and physically preventing the translation process (9).

## 2. Objectives

In this study, in order to obtain an *E. coli* strain with limited amounts of synthesis and released acetate, which will help to expand the life of the lived culture and better production of recombinant protein, we applied this strategy in *E. coli* BL21 (DE3), a broadly employed host strain for recombinant protein production. We synthesized antisense RNA against *pta* and *ackA* genes by using already existing information of metabolic pathways of *E. coli* (6), and studied their effects on acetate release. 

## 3. Materials and Methods

### 3.1. Strains, Media, and Culture Conditions

XL1-Blue sratin (endA1 gyrA96 (nalR) thi-1 recA1 relA1 lac glnV44 F'[: Tn10 proAB+ lacIq Δ (lacZ) M15] hsdR17 (rK- mK+), Tetracyline resistant) (Stratagene) was used for plasmid construction. BL21 (DE3) (F– ompT gal dcm lon hsdSB (rB- mB-) λ(DE3 [lacI lacUV5-T7 gene 1 ind1 sam7 nin5])) (Studier,1986) was used for observation of the antisense cassette effects.

All strains were cultured at 37°C on Luria-Bertani (LB) (Applichem, Germany) complex medium in and then stored on glycerol-stock at -80ºC. Ampicillin (50 μg/mL) was used as a selection marker for strains which have plasmid. For strain creation, cells were grown on LB medium. For physiological characterizations, all strains were cultured on M9 minimal medium (6 g/L Na_2_HPO_4_, 3 g/L KH_2_PO_4_, 1 g/L NH_4_Cl, 0.5 g/L NaCl, 2 mM MgSO_4_, 0.1 mM CaCl_2_) with 10 g/L glucose in shake ﬂasks at 250 rpm. 300 μL of freezer stock was cultured overnight (16 – 18 hours) at 37°C in 50 mL of M9 medium in a 250-mL flask. 

All cultures were cultivated at 37°C, and ampicillin (50 μg /mL) was used as a selection marker for plasmid-accepting strains. To test whether the induction of *asackA *(antisense ackA) under T7 promoter, reduces acetate production, same as past experiment without induction, the acetate concentration was measured in liquid minimal media (M9) containing 10 g/L of glucose. Isopropyl-β-D-thiogalactopyranoside (IPTG) was added at an OD 600 nm = 0.6 with a final concentration of 1 mM. 

### 3.2. Construction of Plasmids With Antisense Cassette

**Figure 1.**
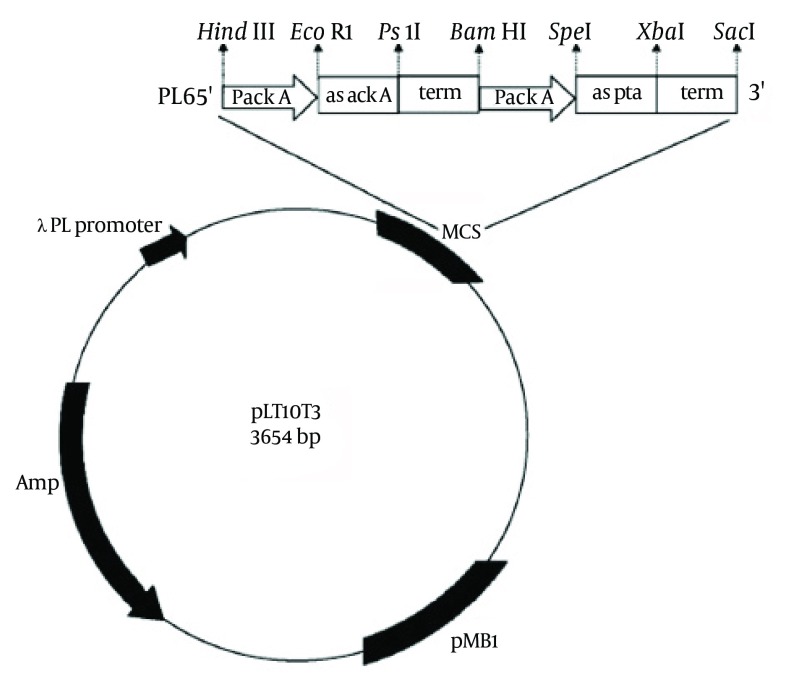
Schematic Diagram of Recombinant Plasmid pL6 is derived from pLT10T3 and contains the antisense genes against both *ackA* and *pta*. Each antisense gene was transcribed under *ackA* promoter and terminated by the pta termination sequence.

Our plasmid, derived from pLT10T3 (BCCM), was constructed to generate the antisense RNAs (asRNA) targeting, acetate kinase (ACK) and phosphotransacetylase (PTA) enzymes. The plasmid named pL6 (Figure 1). Antisense cassette consisted of the *ackA *promoter, antisense to both *pta* and *ackA,* and the termination site of *pta*. All were found in *E. coli* K12 genome (NCBI databank) (10) by use polymerase chain reaction (PCR) amplification with a DNA thermal cycler (AutoQ server thermal cycler, Quanta biotech Ltd). As same as previous promoter sections, expanding from −79 to +21, was chosen to contain an open complex region for transcription initiation, −10 (TATA box); −35, a spacer between these two sections, and the UP element placed in −40 to −60 region of the *ackA* gene (11, 12). The sizes of antisense sequences for *ackA* and *pta* were 141 base pairs (bp) for *pta* and 155 bp for *ackA*, which containing a ribosome binding site (RBS). The ρ-independent termination site of the original *pta* gene was chosen as a terminator region at the end of both antisense sequence (12). All PCR primer sequences of the given cassette are recorded in Table 1. 

**Table 1.** Sequence of Primers (5' to 3')

Target Gene	Sequence of Primers (5' to3')
**Promoter of ackA**	
Forward (for *asackA*)	5َ GA.AGC.TTG.GCA.TAG.ACT.CAA.GAT.ATT.TC 3َ
Reverse (for *asackA*)	5َ AAG.AAT.TCG.TCA.GGG.AGC.CAT.AGA.G 3َ
Forward (for *aspta*)	5َ CCG.GAT.TCT.AGA.CTC.AAG.ATA.TTT.CTT.CC 3َ
Reverse (for *aspta*)	5َ GGA.ACT.AGT.GTC.AGG.GAG.CCA.TAG.AG 3َ
**Antisense ** ***ackA***	
Forward (for *asackA*)	5َ CTG.CAG.TAC.GCT.CTA.TGG.CTC.CC 3َ
Reverse (for *asackA*)	5َ CGG.AAT.TCC.TCT.TCA.CCA.TTT.ACT.GC 3َ
**Antisense ** ***pta***	
Forward (for *aspta*)	5َ TTC.TAG.AGC.TGT.TTT.GTA.ACC.CGC.C 3َ
Reverse (for *aspta*)	5َ CAC.TAG.TAT.TGC.ACG.GAT.CAC.GCC 3َ
**Terminator Region of ** ***pta***	
Forward (for *asackA*)	5َ CTG.CAG.TCT.CTC.GTC.ATC.ATC.CGC 3َ
Reverse (for *asackA*)	5َ AAG.GAT.CCA.TGC.AGC.GCA.GTT.AAG.C 3َ
Forward (for *aspta*)	5َ CCT.CTA.GAT.CTC.GTC.ATC.ATC.GCA 3َ
Reverse (for *aspta*)	5َ GAG.CTC.ATG.CAG.CGC.AGT.TAA.G 3َ

### 3.3. Analytical Procedures 

Cell growth was monitored by measuring the optical density at 600 nm using a spectrophotometer (DU-640; Cecil). Samples for metabolite measurements were collected every 1 hour and spin down in a centrifuge at 9000g for 5 minutes (Biofuge Pico, Heraeus). The supernatant was retained and stored at -20°C until assayed. The glucose concentration in the medium was measured using the GOD-PAP kit (Biolab, France). Acetic acid concentrations were determined with enzymatic experiment kit (Boehringer-Mannheim, Darmstadt, Germany). Relative activities of PTA and ACK were measured using the Brown et al. protocol (13). Cell extracts were provided in this way: 50 mL of cultured cell broth at mid-log phase (approximately 1.0 of OD600) from given Erlenmeyer were collected at 5000 rpm for 10 minutes at 4°C and rinsed two times with 30 mL washing buffer (10 mM MgCl_2_, 1 mM EDTA, 10 mM sodium phosphate (pH 7.5)). 

The cells were suspended in 1 mL of washing buffer, sonicated for 5 minutes on ice, and centrifuged at 13000 rpm for 30 minutes at 4°C. Protein concentration in the supernatant, as crude extract, was measured by the Bradford method by using the Bio-Rad dye reagent with bovine serum albumin as a protein standard. ACK assay performed in the mixture containing, 15 mM malic acid, 22.5 mM NAD+, 3.75 mM CoA, 20 μg/mL citrate synthase, 20 μg/mL malate dehydrogenase, 1 U/mL PTA, 60 mM ATP, 225 mM Tris-HCl (pH 7.6), 12.5 mM sodium acetate and 4.5 mM MgCl2. PTA assay was performed in the reaction mixture containing 15 mM malic acid, 22.5 mM NAD+, 3.75 mM CoA, 20 μg/mL malate dehydrogenase, 10 mM AcP, 20 μg/mL citrate synthase, 4.5 mM MgCl_2_, 225 mM Tris-HCl (pH 7.6). The reaction initiated by adding crude extract equal to the protein concentration. Activities of PTA and ACK were determined by observing the absorbance at 340 nm (ε340 = 6.22 mM−1 cm−, 11 U= 1 μmol/min).

### 3.4. Quantitative Analysis of mRNA Transcription by RT-PCR

**Figure 2.**
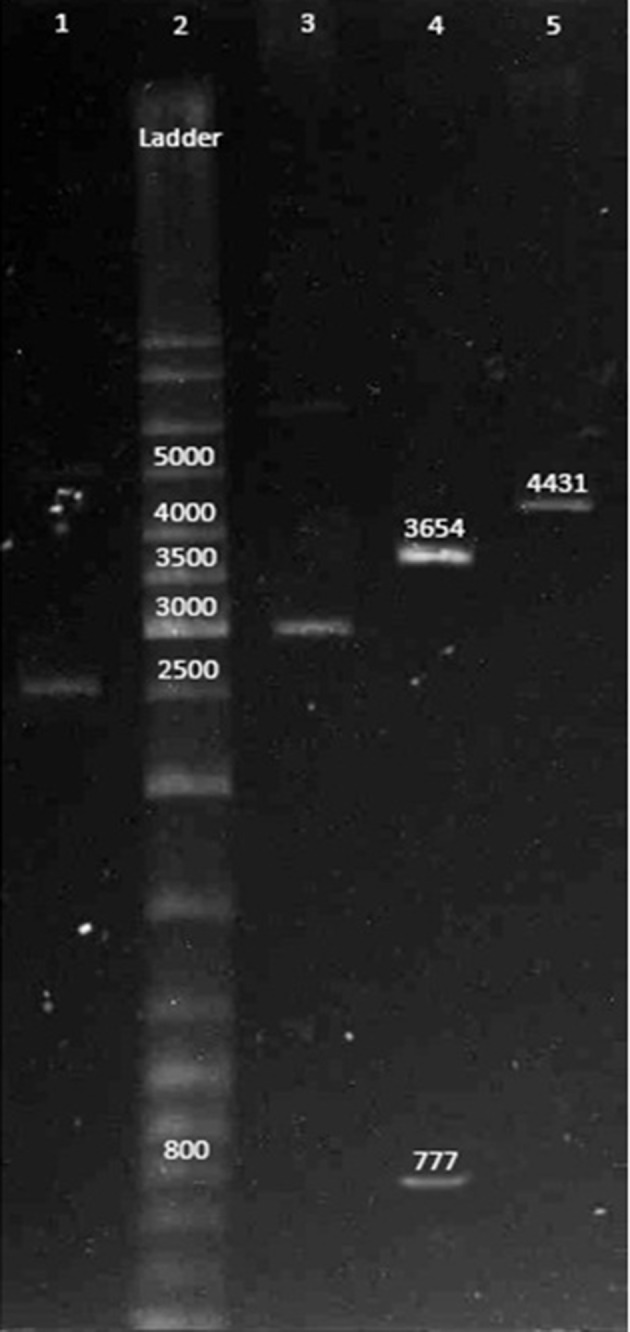
Electrophoresis Diagram of Recombinant Plasmid Lane 1: pLT10T3; Lane 2: ladder; Lane 3: pL6; Lane 4: pL6 digested with *HindIII* & *SacI* and released 777 bp antisense cassette; Lane 5: pL6 digested with *BamH1.*

Extraction of total RNA was achieved according to the manner of Karunakaran and Kuramitsu (14). All cultures (5 mL) harvested at mid-log phase (~1.0 of OD 600). Samples were treated by an alkalilysis method and rinsed with ethanol. Following dried outing, the RNA pellet was dissolved in DEPC treated double-distilled water and then treated with DNase I (Fermentas, Lithuanian) to eliminate the genomic DNA. Any remaining DNase was inactivated at 65°C for 10 minutes. The RNA in each sample was measured by absorbance at 260 nm under UV–visible spectrophotometer (1 absorbance unit = 40 μg of RNA) (Roche Applied Science Lab Faqs, Germany). Quantitative analyses of mRNA transcription of *pta* and *ackA* genes were achieved by using reverse transcription (RT) PCR. The quantity of total RNA chosen as a template for RT-PCR was 5 μg in each reaction for the first strand cDNA synthesis by reverse transcription. To keep away from effect of quick decay of bacterial mRNA from the 5' end, each primer was designed to amplify a section of the gene in the later part of complete mRNA (Figure 2). 

Reaction compositions were total RNA (5 μg), 3'-end primer (1 μM), 40 U/μL RNase inhibitor (0.5 μL), 5 U/μL AMV reverse transcriptase (1 μL), 10 mM dNTP mix (1 μL), 10x RT buffer (2 μL), and DEPC-treated double distilled water to a total volume of 20 μL. The reaction was performed in a thermal cycler at 48°C for 50 minutes, then remaining reverse transcriptase was inactivated at 94°C for 2 minutes; 2 μL of RT product was utilized as a template for PCR used for quantitative analysis. The PCR reaction was achieved in the following reaction mixture: RT product (2 μL), forward and reverse primer (1 μM), 10 mM dNTP mix (1 μL), Taq polymerase (2 U), 10x buffer (5 μL) and distilled water to a total volume of 50 μL. 

Denaturation at 96°C for 5 seconds, annealing at 60°C for 5 seconds, and extension at 68°C for the 28 seconds for *ackA* and 60 seconds for *pta* performed. For amplification of desired fragments the cycles repeated 30 times for both *ackA* and *pta*. Reverse transcription was also performed on the total RNA without RT for inspection of genomic DNA contamination. The image of the gels was analyzed using a gel documentation system (Opticom500, Isogen life science). Intensities of bands were measured by total lab image analysis software (Isogen life science). All primers applied in RT-PCR for quantitative analysis of mRNA transcription are shown in Table 2. 

**Table 2.** Primers Used in RT-PCR for Quantitative Analysis of mRNA Transcription of *ackA* and *pta*

Target Gene	Sequence of Primers (5' to 3')
***ackA***	
Forward	5'CGATGCAGTAAATGGTGAAGAG 3'
Reverse	5' ATCAGCGCAGTGTAGGCAC 3'
Forward	5' AGGAAGCGGCTTTAGGTG 3'
Reverse	5' ATCAGCGCAGTGTAGGCAC 3'
***pta***	
Forward	5' CCGTATTATTATGCTGATCC 3'
Reverse	5' GCTGTACCGCTTTGTAGG 3'
Forward	5' GTGCTGATGGAAGAGATCG 3'
Reverse	5' GCTGTACCGCTTTGTAGG 3'

## 

## 4. Results

### 4.1. Plasmid Containing the Antisense Cassette

All antisense cassette segments were amplified by PCR using the chromosomal DNA of *E. coli* K12 as the template. Initially, the PCR products were cloned into pBluescript SK + (strata gene) according to Figure 1 and then complete cassette was subcloned into pLT10T3. Plasmid containing antisense cassette, pL6, is shown in Figure 2. 

### 4.2. Antisense Down-Regulation of Target Transcripts and Enzyme Activities

**Figure 3.**
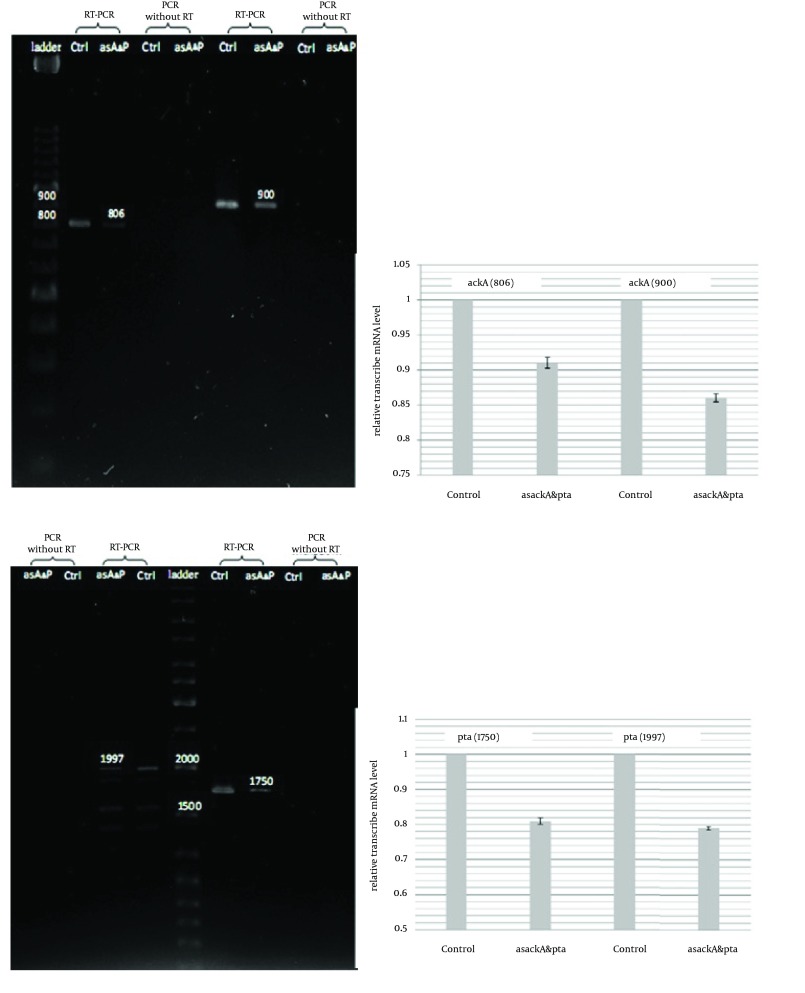
Quantitative RT-PCR Analysis of Antisense Cassette Function (a) RT-PCR on the region after the hybridization zone of asRNA and region involving the hybridization zone of *ackA*. (b) RT-PCR of the region after the hybridization of asRNA and region involving the hybridization zone for *pta*. Reverse transcriptions were carried out 3 times on each total RNA sample, prepared from separated series of cell cultures. Two PCRs carried out for a template got from each reverse transcription. DNA band intensity was analyzed, and the values were averaged to calculate relative transcribed mRNA levels of *ackA* and *pta* from the antisense-regulated strain compared to the control. Ctrl, control (pLT10T3); as A and P, asackA and aspta (pL6)] are PCR products after reverse transcription; other lanes are the control PCR samples without reverse transcription to check for any contamination of genomic DNA fragment in the PCR products. The error bars represent the standard errors from repeated RT-PCRs for each gene.

Quantitative RT-PCR was used for measuring the transcription levels of *pta* and *ackA* shown in Figure 3. 

**Figure 4.**
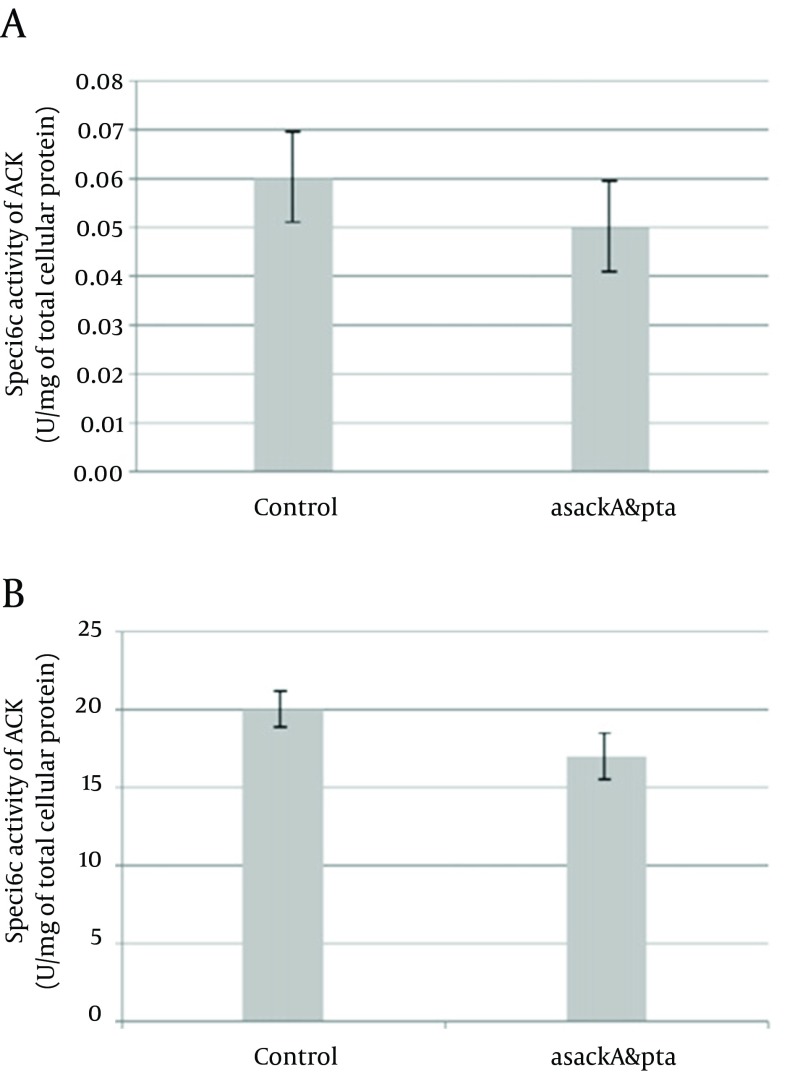
Specific Activities of (a) Acetate Kinase (ACK) and (b) Phosphotransacetylase (PTA) *in E. coli BL21* Strains Harboring pLT10T3, and pL6 (asackA and aspta). The error bars represent the standard errors from repeated assays of two separated culture series.

These experiments conducted three times for each total RNA sample obtained from separated series of cell cultures. Two kinds of PCR fragments were amplified, the first fragment was spanned from the next-to-the-hybridized zone by antisense mRNA to near the ending of transcription site, and the second one was a longer segment from an overlapping area with the hybridized region by antisense mRNA to almost the ending of transcription. PCRs were done two times for each reverse transcription. The DNA band concentration was analyzed and the amounts were normalized to determine relative transcribed mRNA levels of *pta* and *ackA* from the antisense-regulated strain and compared to the control. Similar to the report of Kim and Cha (8), for both amplified *pta* and *ackA* fragments, the asackA and aspta regulated ones showed quite minor levels of *ackA* (9-14%) and *pta* (19 - 21%) mRNA than the control strains (Figure 3 b, 3 c). We also found down-regulation activity of antisense cassette on enzymatic activity of ACK and PTA. We observed relatively decreased activity of each enzyme (on average, 15% for PTA and 17% for ACK) consistent with each antisense regulated strain (asackA and aspta) compared to the control harboring the primary plasmid pLT10T3 (Figure 4). 

### 4.3. Influence of Antisense Expression on Cell Physiology

**Figure 5.**
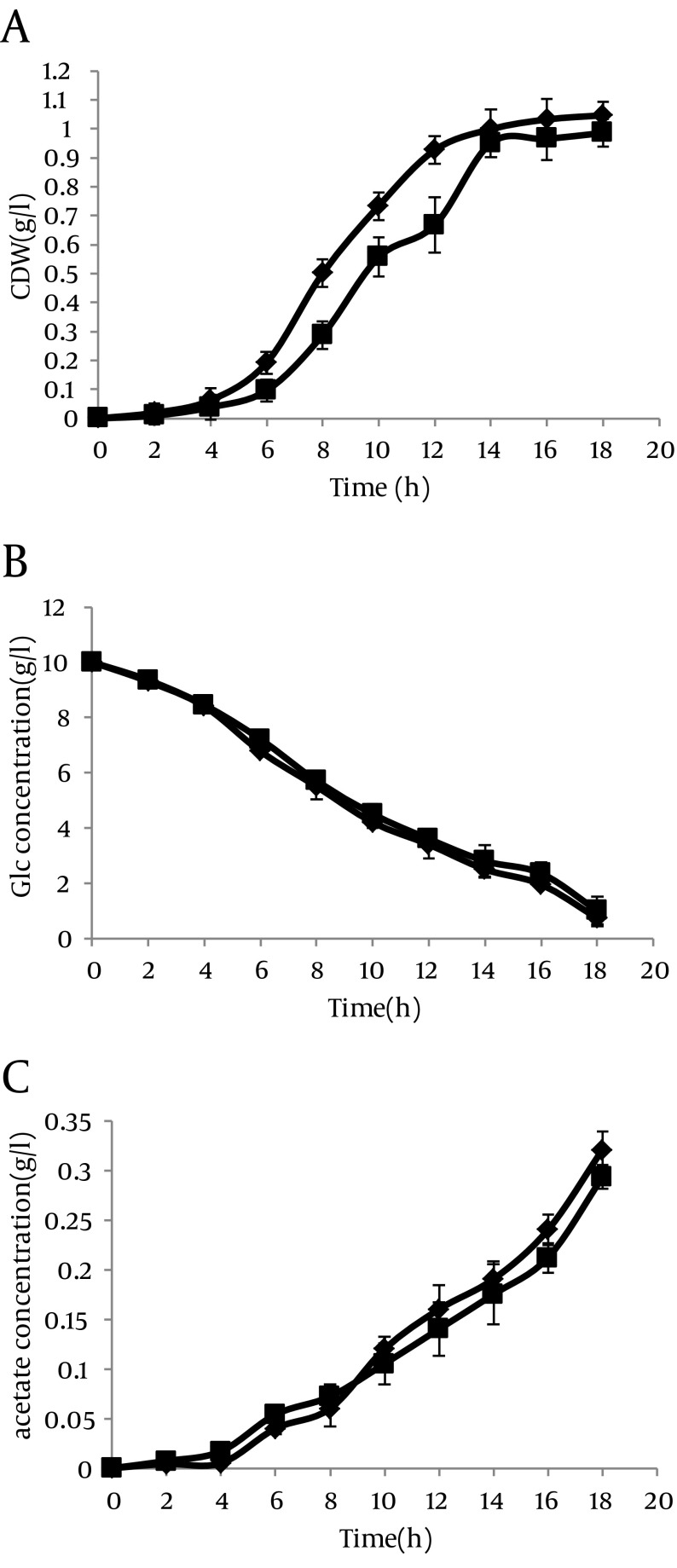
Time Profiles of Cell Growth, Residual Glucose Concentration, and Acetate Concentration in *E. coli BL21* Time profiles of (a) cell growth, (b) residual glucose concentration, and (c) acetate concentration in *E. coli BL21*(DE3) Strain harboring(■) pLT10T3, and (♦) pL6 (asackA & aspta). Cells were grown in M9 plus glucose media at 37°C. Values are the average of triplicate experiments.

**Figure 6.**
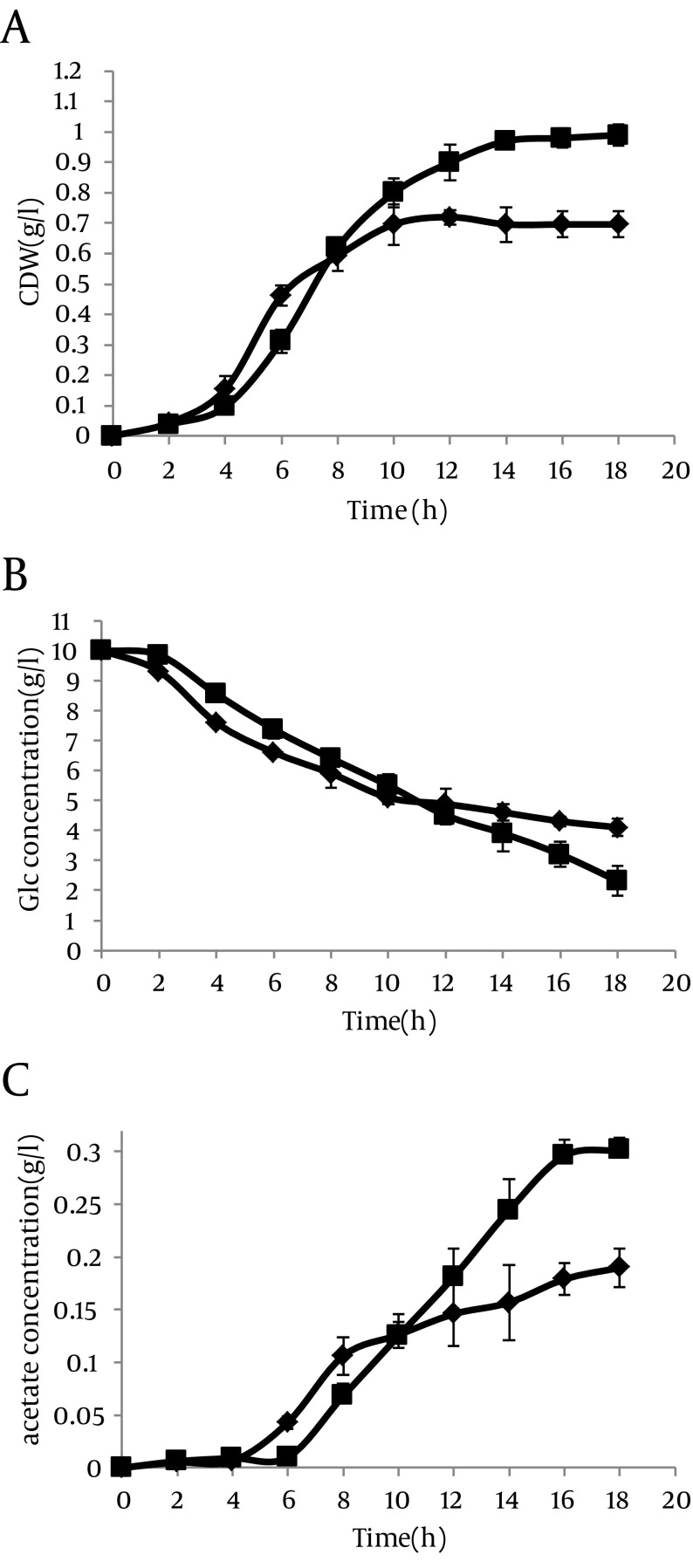
Effect of ackA-pta asRNA on (a) Cell Dry Weight, (b) Residual Glucose Concentration, and (c) Extra-Cellular Acetate Concentrations for Each Transformant Strains harboring pLT10T3 are shown by (■), and pL6 (asackA and aspta) by (♦). Cells were grown in M9 plus glucose media with IPTG induction at 37°C. Cultures harvested during the logarithmic phase of growth in the presence of IPTG.

The growth curves and utilization rates of the individual carbon resource (glucose) were obtained by measuring the samples collected from the cultures of *E. coli* BL21 (DE3) transformed with the recombinant plasmid, pL6 (asackA and aspta) and compared with the control strain containing the origin plasmid pLT10T3 without and with IPTG (1 mM). In absence of IPTG, cells with antisense cassette showed higher growth rate compared to control bacteria (Figure 5 a), but this speed decreased gradually when IPTG was added to the culture media (Figure 6 a). In addition, the glucose consumption pattern during growth curve showed an increase in both strains with and without IPTG (Figures 5 b, 6 b). The acetate production level in absence of IPTG showed that even though the antisense-regulated strains showed fairly minor amounts of *pta* and *ackA* mRNAs, the secreted acetic acid level was not decreased compared with the control, which confirms the data reported by other researcher (8) (Figure 5 c). 

**Figure 7.**
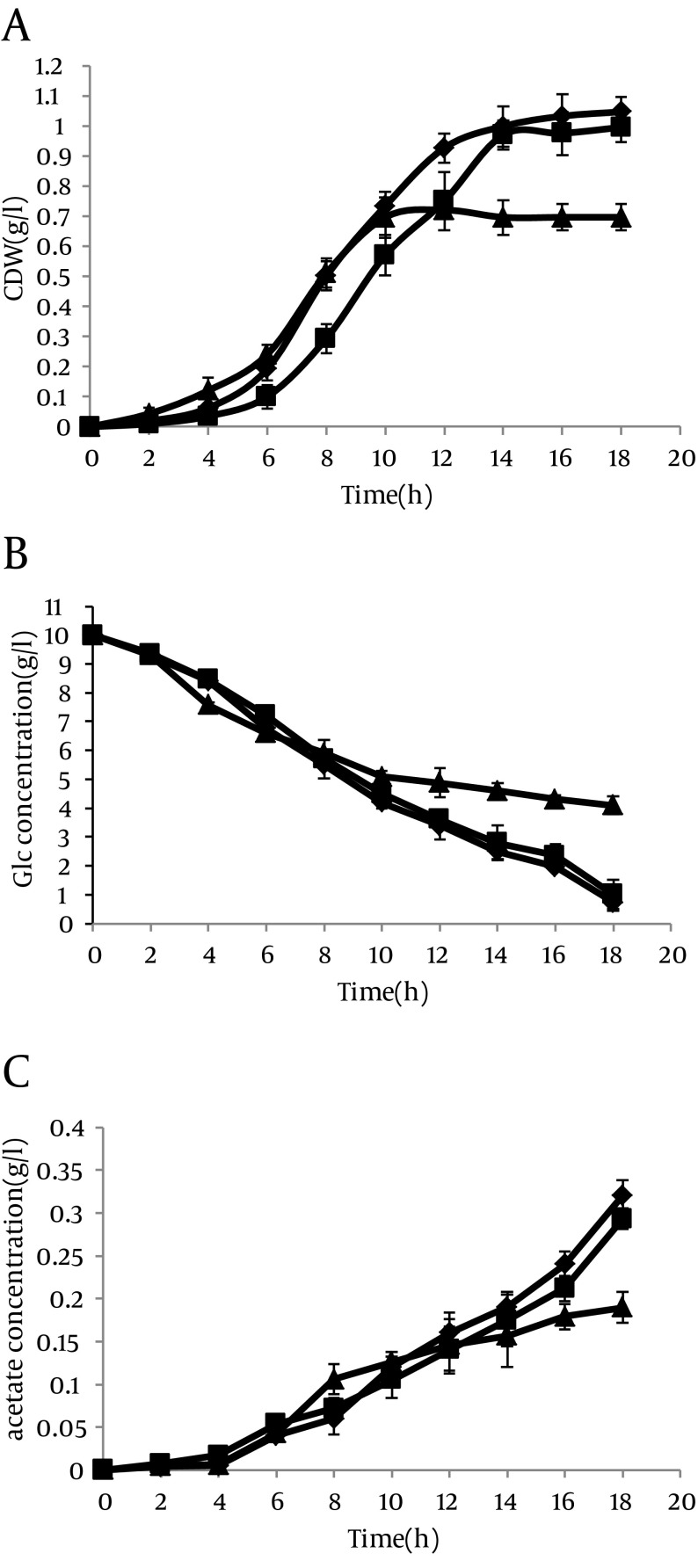
Effect of ackA-pta asRNA on (a) Cell Dry Weight, (b) Residual Glucose Concentration, and (c) Extra-Cellular Acetate Concentrations for Each Transformant Strains harboring pLT10T3 are shown by (■), and pL6 (asackA and aspta) by (♦), and pL6 induced by IPTG is shown by (). Cells were grown on M9 plus glucose media at 37°C. The second cultures harboring pL6, harvested during the logarithmic phase of growth in presence of IPTG.

However in the presence of IPTG, the results were completely different. In this case, acetate excretion in strain with antisense cassette decreased clearly 2 hour after induction (Figure 6 c). The mentioned parameters have been compared in cells without antisense cassette and those with antisense cassette in absence and presence of IPTG. The results have been shown in Figure 7. 

## 5. Discussion

Previous studies showed that, in mixed-acid fermentations, the acetic acid excretion happens anaerobically (15). In addition, when bacterial growth takes place on excess glucose condition (or other highly assimilable carbon sources) that inhibits aerobic respiration, it happens aerobically (16, 17), a phenomenon identified as the bacterial Crabtree effect (18). Because high acetic acid concentration can prevent the growth and foreign protein production (19-23), therefore a variety of operational approaches have been previously suggested and checked to decrease the level of acetic acid accumulation (2, 22, 24-33).

In this study, we applied recently used antisense RNA approach for down-regulation of ackA-pta pathway. We performed this method in an extensively used host strain, *E. coli BL21* (DE3), for production of foreign proteins, that no study has been conducted. We could observe that bacteria growth in improved mass in presence of the antisense cassette might be an indicator of better growth condition and prevent the cell death (Figure 5 a). At the same time, more acetic acid was produced by the fast growing antisense-regulated strains (Figure 5 c). 

These consequences were like to those observed on different strains reported by Kim and Cha (8) and Nakashima, et al. (34), since the phase of cell growth and physiological state affect the expression of ackA and pta (35-37), it was found that if whole transcribed mRNA level were higher, as a result of elevated cell densities, partial repression of ackA-pta pathway by the antisense method could not decrease the acetic acid excretion even in antisense-regulated strains (8). In addition, it should be mentioned that *E. coli* has at least two paths for producing acetic acid (POXB and ACK-PTA pathways) (34, 38). Recently, it has been demonstrated that a *poxB* knockout strain considerably reduced the acetic acid production when the strain was subjected to oscillatory oxygenation (39). Also, in another research, it has been observed that overexpression of pyruvatecarboxylase in a *poxB* mutant caused an 80% decrease in acetic acid production (40).

However, this result was obtained when a weak promoter was used in antisense cassette. Thus we studied specific parameters when the T7 promoter, located upstream of antisense cassette, was induced by IPTG. In this case, antisense regulated cell growth rate was diminished clearly in comparison with control experiments and the cells were entered to the stationary phase with lower cell dry weight (CDW) (Figure 6 a). Similar results with lower intensity were lately reported studies on other strains of *E. coli* (34). Acetate concentration also decreased in induced antisense regulated cells (Figure 6 c). A recent report elevates the possibility of acetic acid formation by providing the TCA cycle enzyme 2-ketoglutarate dehydrogenase (KGDH) with CoASH primarily allows more speedy growth to higher cell densities (41). Therefore, it can be said that acetic acid pathway has more important role in cellular physiology than those expressed in earlier reports. In addition, an ackA-pta deletion mutant excretes fewer acetic acid than wild type cells by reason of incomplete carbon ﬂux to acetic acid (27). 

Although little repression of *pta* and *ackA* genes by antisense could not decrease acetic acid formation, with high induction, ackA-pT7 asRNA effects can be enough for imitate the phenotypes of the simple mutants. Finally, we have shown that this cassette changes the release of acetate and the growth rate of *E. coli* BL21 (DE3), and then it can be applied for controlling the recombinant protein production by this host. 
